# Perception, Acceptance, and Challenges Regarding a New Framework for Security, Safety and Health Enforcers: A Qualitative Study in Malaysia

**DOI:** 10.21315/mjms-12-2025-913

**Published:** 2026-08-31

**Authors:** Hassan Ismail, Emilia Zainal Abidin, Irniza Rasdi, Kulanthayan K.C. Mani, Phaik Kin Cheah

**Affiliations:** 1 Department of Environmental and Occupational Health, Faculty of Medicine and Health Sciences, Universiti Putra Malaysia, 43400 UPM Serdang, Selangor, Malaysia; 2 Department of Community Health, Faculty of Medicine and Health Sciences, Universiti Putra Malaysia, 43400 UPM Serdang, Selangor, Malaysia; 3 Department of Public Relations, Faculty of Arts and Social Science, Universiti Tunku Abdul Rahman, Jalan Universiti, Bandar Barat, 31900 Kampar, Perak, Malaysia

**Keywords:** compliance, integrated enforcement, legislation, roles, SSHE framework

## Abstract

**Background:**

Non-compliance with legal requirements related to occupational safety and health (OSH) among workers in Malaysia remains widespread. Contributing factors include insufficient enforcement workforce. Given the large number of security personnel (SP) and the overlapping nature of responsibilities, there is potential to integrate security roles with OSH functions. This study aims to identify the perception, acceptance and challenges of security-related stakeholders on the possible integration.

**Methods:**

An exploratory qualitative research was performed in Klang Valley, Malaysia. Management representatives from various security and safety organisations were invited to participate in focus group discussions (FGDs). Data were analysed using framework analysis. Voice recordings were transcribed, translated and coded using NVivo v14, and findings were interpreted based on organisational readiness for change theory.

**Results:**

Eleven representatives participated in two FGDs. The integrated enforcement concept was preferred for adoption by both groups. Although the majority of security guards are Malaysians, many foreign guards are found in commercial and private sector premises, auxiliary police and the Malaysia Volunteer Department (RELA) were limited to Malaysians. The current security training curriculum has minimal basic OSH content. There is a need to integrate OSH into security training modules while benchmarking OSH courses. Enablers consisted of the need for training and new basic modules for SP, leadership, resource optimisation, minimum qualifications and the latest technology.

**Conclusion:**

The integration of functions is favoured by management representatives of security and safety organisations. Challenges include limitations in legal requirements, and enablers focus on the need for changes in the curriculum for training and leadership support. The findings of this study support the goals of SDG 8 and OSHMP30.

## Introduction

There has been a concerning increase in workplace and commuter accidents due to high rates of non-compliance against occupational safety and health (OSH) legislation by workers in Malaysia. Healthcare and compensation costs have risen from MYR 2.4 billion in 2014 to MYR 5.7 billion in 2023 (1). Malaysian occupational accident rates have consistently been higher than those in other countries (e.g., the UK, Japan) (2). OSH-related legal noncompliance has been reported by authorities, and 30% to 50% of industrial accidents are caused by small and medium-sized enterprises (SMEs) (3). It may be interpreted that adherence to OSH regulations is challenging when financial resources are limited for smaller-scale industries (4). Although Malaysia has strong OSH rules, authorities may not be able to consistently ensure implementation of legislation (5). Safety and health officers (SHOs) are tasked with supporting employers to ensure OSH standards in the workplace, and statistics show that 5,962 officers serve 16.69 million workers (6, 7). With 1.6 million companies and 9.2 million enterprises (8), it is reasonable to expect authorities to face challenges in enforcing OSH legislation, particularly for SMEs (9, 10).

In Malaysia, there are several types (and numbers) of security personnel (SP), namely RELA (3.1 million), auxiliary police (AP; 0.029 million) and security guards (0.2 million) (11, 12). By definition, security focuses on preventing spying and thievery while establishing a secure workplace (13). Functions of SP include risk management, data and asset loss prevention, including technology security, and deterring criminal activity (11). While these functions are not the same as OSH, there are overlaps such as duties related to patrolling and promoting safety by using personal protective equipment (PPE) (11). The large number of SP available can be leveraged by merging security roles with those of OSH because security has substantial enforcement authority.

The hypothesis of merging security roles with OSH-related functions, such as access control, PPE, inspection, emergency evacuation, first aid, investigation, incident reporting, surveillance and security camera monitoring, could benefit the industry (11). Compliance would ensure worker safety, protect businesses from fines, increase revenue, build client trust, and simplify policies and processes (14). In addition, technological advancements can significantly change the nature of safety work practices and allow more temporal and spatial opportunities for the merging of roles and functions (15).

Based on the possibility of the merging of roles, this study sets out to explore the acceptance and challenges in integrating security and OSH roles, aiming to develop a new framework for security, safety and health enforcers (SSHEs). The findings of this study support the governance of OSH at the workplace and align with Malaysia’s OSH Master Plan 2030 (OSHMP30), focusing on policy upholding and enforcement transformation in addition to supporting Malaysia to achieve its UN Sustainable Development Goals 8 and 12 (16) and economic growth under the economic planning unit, with a focus on goals 8, 9 and 17 (17).

## Methods

In this exploratory qualitative study, a framework approach based on organisational readiness for change theory (ORCT) (18) was applied when no standard framework integrating the roles of security and safety was found. This study used deductive approaches to predetermine initial codes and inductive approaches to determine newly emerging themes from focus group discussions (FGDs) to develop a new framework (19–21).

### Study Setting

Fourteen agencies were invited to attend FGDs as stakeholders, with 28 representatives nominated for a study in Klang Valley, Malaysia. Participants were selected by their organisation using the researchers’ specified inclusion criteria; most were government officers, senior managers, senior consultants and non-governmental organisation (NGO) officers from reputable Malaysian organisations. Participants were required to have worked for at least three years as either contract or permanent staff in organisations. Furthermore, they must have had roles related to primary enforcement. Organisations were sent a letter outlining inclusion criteria to solicit involvement; they used these criteria to provide suggestions for participant recruitment.

### Sampling Strategy

The study used homogeneous sampling to ensure similar backgrounds. This is to ensure that participants can engage well in FGDs and can consider different viewpoints, ensuring open discussion (22).

### Participant Selection

The researchers recruited 11 participants between July and November 2023. All participants provided written consent before voluntarily participating. They were briefed about FGD guidelines and conversation confidentiality (22).

### Data Collection Methods

FGDs were used to collect data to conduct process triangulation, incorporating validity elements such as member verification and researcher reflexivity (22–25). The study was conducted in a private meeting room facility at Universiti Putra Malaysia. Each FGD session lasted 90 min. Participants in FGDs represented both men and women, split into two groups. The researchers managed pre-selection preparation, script familiarisation, group dynamics, seating arrangements, equipment and meeting documentation. All conversations were audio-recorded with consent. FGDs were facilitated by a moderator and note-taker. The researcher began FGDs with open-ended, probing questions to encourage conversation in the local language (24). The framework approach included transcription, familiarisation, identification of the theme framework, coding, charting, and developing an analytical framework. Applying the analytical framework, charting data into the framework matrix and interpreting all data underwent iterative processes (19–21). The researchers used reflexivity observation and notes to evaluate data and decide which questions to look into further (22–25).

### Topic Guide

This study used a topic guide consisting of semi-structured questions (24). Example questions were “Can you suggest any modifications or adjustments to the proposed integrated model that might make it more effective or efficient within your organisation’s context?” “How might changes in the organisational setting and external environment affect the innovation sustainability of this new option enhancement?” and “How will the new option change the organisation and maximise all resources to improve and deliver great service to the public?”

### Data Analysis

The study used voice recordings as the main data source. Anonymised voice recordings were transcribed using the verbatim approach and were translated from Malay to English by professional services. The transcripts were validated by the research team to maintain the integrity of transcription and translation. Researchers analysed the transcripts, and coding framework analysis was performed using NVivo v14 (26). Data were analysed using the framework approach, which involved systematic coding based on existing theory (19–21). During data coding, the researcher highlighted pertinent phrases and concepts to identify themes that emerged. Each focus group participant’s input was “constantly compared” for similarities. The comparisons and commonalities led to the emergence of conceptual categories.

## Results

### Statistics on the Demographics of Key Informants

Key informants (KIs) consisted of Malay (90.9%), and their designation included government officers, deputy chief executives, senior consultants, general managers and NGO officers. On average, both groups had over 23 years of experience. Table 1 presents sociodemographic data of 11 participants.

As a result of the framework approach coding, 17 initial codes were pre-identified through literature review and existing theory frameworks, 20 codes in total, with three new emergent codes discovered. These were grouped into 14 subthemes, main themes, three emergent categories and themes mapped closely to the key constructs of ORCT. Elements of change commitment and efficacy are central to Weiner’s theory (18). Change commitment highlights the importance of employees valuing necessary change innovations and considers whether stakeholders believe change is worth their time and resources. Change efficacy is influenced by how organisational members evaluate implementation capability, which emphasises the importance of perceptions regarding a team’s ability to implement change. This framework allows for exploration of integrated job descriptions, legislative power sources, leadership and other relevant issues. A notable opportunity for integration arises when the safety framework of each organisation is understood. Figure 1 outlined the framework analysis for the coding performed.

### Acceptance of the Concept

Based on FGDs, both groups were willing to adopt the new framework of security and OSH. Specifically, one of the groups mentioned that the idea was beneficial and believed that integration could occur. The group also mentioned that the industry would find the integration useful at the operational level:

“It can be implemented actually.” (FGD 1)

“I prefer to integrate both; highly beneficial integration can occur, market needs this tool.” (FGD 2)

### Perception on Responsibilities Aligning with Roles

There is also the perception of combining work and giving security guards additional responsibilities as logical because they possessed enforcement authority:

“Section 5(1)(a) of Act 752 grants the government power to assist other departments during COVID-19.” (FGD 1)

“Enforcer must come from security because they have authority; current reporting structure oversees both security and safety.” (FGD 2)

### Challenges

As it is accepted that certain SPs are provided with the authority to impose fines, KIs mentioned the need to consult all stakeholders, including raising public awareness, to provide adequate information before OSH enforcement. This is to prevent non-acceptance and issues, especially if related to the issuing of fines.

“Knowledge is not just for enforcers and general public; if someone doesn’t know, and they fine, it can be scary.” (FGD 1)

“In the regulations, it’s not present.” (FGD 2)

### Foreign Workers

Among the challenges noted in FGDs was the limitation of foreign security officers as enforcers. KIs emphasised the need for legislative change to include health and safety requirements in security laws, as OSHA 1994 does not mention security duties and the prohibition of foreign enforcers, while safety officers are all Malaysian.

“Security guards, many of them are foreigners.” (FGD 1)

“In OSH legislation, there is no mention of security personnel. Safety officers are local. Law prohibits hiring foreigners.” (FGD 2)

### Enablers of Legislation, Organisations and Workers

From the framework analysis, several factors which resembled enablers were identified, and these factors have been mentioned as crucial to ensuring the hypothetical framework where security is integrated with OSH.

### Support and Resources Required for the New Framework

For security guards, KIs mentioned that the enforcement body requires resources to improve guard rooms, to provide new modules and training centres in order to enable them to perform tasks supporting compliance with OSHA 1994.

“Training centres not enough.” (FGD 1)

“Security guard room.” (FGD 2)

### Significance of OSH Training

The group emphasised the need for OSH training to increase knowledge specifically for SP, highlighting that the roles of security and safety were different. KIs commented that the certified security guard (CSG) training modules are limited to 4 h and cover only a small part of OSH knowledge. In addition, the training has a minimal practical aspect and exposes workers to risks.

“It is advisable that all SP are provided with basic [OSH] knowledge; they didn’t practise, they don’t know the importance. Their health needs to be taken care of; there’s a risk.” (FGD 1)

“Basic modules such as CSG is only four hours in six days, [there is only] basic OSH.” (FGD 2)

KI’s emphasis on basic OSH training for SP may increase costs and training days:

“Training requirements may change, OSH modules should be added; crucial.” (FGD 1)

“The expense…must pay for more training days.” (FGD 2)

Different non-compliance enforcement settings were noted by KIs, such as the inability of AP to impose penalties for cafeteria smoking because penalties by law apply to authorities such as council officers or health departments:

“Penalties for smoking in canteens, but the local office or health department issues fines, not the AP.” (FGD 1)

### Skills Required and Minimum Qualifications

KIs suggested tailoring required skills for new roles, focusing on skill certificates and advanced courses for subject matter experts, and using national occupational skill standards (NOSS) for assessment to enable enforcers to perform new duties:

“Have a certificate confirming their proficiency; skill certifications at levels 1, 2 and 3 to be designated subject matter experts.” (FGD 1)

“Comparing with the existing, NOSS can determine the appropriate duration and conduct a training needs analysis.” (FGD 2)

KIs underlined the prerequisites for future enrolment for the new framework in which a minimum qualification of higher secondary education in addition to work experience should be considered:

“A minimum qualification SPM and more than three years of work.” (FGD 2)

Because OSH-related knowledge is covered in just 4% of SP training, KIs suggested that SP and agencies yet to implement OSH training have a newly developed training curriculum:

“Basic training for safety and health can be extended to other enforcement authorities.” (FGD 1)

“Extending training to authorities.” (FGD 2)

### Job Promotion

KIs emphasised the importance of job promotion, in which AP and RELA have defined ranks in legislation, while this is not the case for security guards, and such consideration could be extended in the future.

“The rank for security guards does not exist in legislation; RELA and AP, they have a rank.” (FGD 1)

### Fixed Penalties or Summons

KIs suggested that guards misuse penalties, while RELA and AP have the authority to impose fines, as private sector penalties are determined by management, unlike the public sector:

“Authority to issue summons (fines) may be to AP and RELA, but the potential misuse of power for security guards raises concerns.” (FGD 1)

“Currently, enforcement is initiative; house rules, policies and self-regulation to establish a penalty system are powers given by the management, not from the government.” (FGD 2)

KIs suggested that government agencies should avoid using summons for penalising workers, preferring warning letters or penalties. Public summons pose challenges but may be implemented in the private sector:

“If an outsider commits an offence, where the summons revenue will be parked? Summons are not practical; issuing warning letters or penalties for internal workers.” (FGD 1)

“AP, security enforcers will impose penalties; for example, if a tanker spills oil, a fine of MYR 1,000.” (FGD 2)

### Use of Technology

KIs emphasised that the new framework can be applied with the support of technologies like artificial intelligence (AI):

“With AI features, the first is the costing.” (FGD 1)

“If we delve into AI, it has already been implemented outside the country. Now, CCTV is used. If someone is not wearing a helmet, it will trigger a ‘tut tut tut’ sound and charge them immediately.” (FGD 2)

### Leadership

KIs suggested the creation of a new ministry (29) or department to improve workflow and encourage integration through self-regulation:

“A new ministry or department dedicated to this only.” (FGD 1)

“Integration is all about as far as practicable. A leadership structure willing to accept self-regulation.” (FGD 2)

KIs emphasised the significance of leadership in implementing this model, with the private sector’s top management leading the integration:

“Where it’s unclear if security should take the lead. For instance, the head of security leads safety; below is a safety team. In some places, safety is positioned at the top, while security is placed below.” (FGD 2)

KIs suggested converting cost centres to revenue centres as companies compete and generate revenue:

“Currently, the proven existing penalty system works; money will go into the finance department. Safety and security, it is an expenses department; this strategy, see our income.” (FGD 2)

### Multiskilling/multifunctionality

KIs highlighted the necessity of multi-level skills in companies based on their business nature, a certified person in charge (PIC) and enhanced enforcement team competencies through specialised training:

“Employer needs to appoint a PIC, [who is] recognised and has a certification.” (FGD 1)

“Competencies of AP as firefighters, paramedics and competencies developed through integration and training, similar for high-rise buildings.” (FGD 2)

### External Environment

KIs highlighted challenges organisations face in employing foreign SP (FSP) for OSH fines. The main point mentioned was managing complaints to avoid reputational damage and public dissatisfaction:

“FSP, if they want to enforce it, the public may not accept it; can be implemented but with caution.” (FGD 1)

“We have FSP; they enforce it limited to house rules.” (FGD 2)

### Development of a New Framework

Both groups agreed on necessary role modifications to align with leadership direction, manage stakeholder perceptions, and link responsibilities to regulations and policies, addressing resource shortages and clarifying security versus safety roles to prevent ambiguity. They suggested that enforcing OSH laws is vital for boosting awareness, necessitating precise job descriptions, organisational structure and legal considerations that incorporate OSH responsibilities through enforcement. The need to create new key performance indicators (KPIs) for SSHEs is highlighted by the noticeable absence of enforcement:

“Everyone is aware of OSH; currently, only some people know.” (FGD 1)

“Clear job scope, agreement included specific responsibilities of SP’s duties tied to OSH enforcement, sustainability is observable; key performance indicators need to be established.” (FGD 2)

KIs supported work changes, mini medical rooms, structured training and organisational change readiness to enhance OSH knowledge, community awareness, job scope, KPIs and sustainability:

“Lack of equipment…, boundaries apply, developing standard operating procedures and standing operating orders is crucial; safety black book does not emphasise occupational health.” (FGD 1)

## Discussion

The study examines the integration of SP in Malaysia, revealing limited health and safety training due to prioritising security protocols compared with OSH curriculum (27). KIs agreed that integrating OSH responsibilities into security enforcement is viable, but must exclude armed forces, domestic workers and ship workers since their laws differ (2). Group 1 proposed a new coordination organisation, replicating the Malaysian office of the chief government security officer (28).

The implementation of the SSHE model requires organisational readiness, adaptation of employee structures, legislation, security policies, standard operating procedures, company-specific regulations, training costs and new KPIs for enforcement effectiveness, with GCE O-level qualifications suggested by KIs (29). Organisational changes are guided by ORCT (18). The SSHE model, which acts as a fully integrated enforcement framework, handles security, safety and health compliance challenges in the industry (30). It demonstrates preparedness for prospective changes in the organisation based on data synthesis (Figure 2).

The study emphasises the significance of acquiring OSH skills for enforcement and compliance, supporting the creation of a new OSH standard training module for SP basic training (2, 31), enhancing their ability to identify workplace risks (32). The SP roles are similar to campus police and community support officers (CSOs) (33, 34).

SP training focuses on security protocols, with minimal OSH-related topics. To improve OSH practices, OSH courses should be tailored to different organisations and use Malaysian Skills Certificate levels (35), providing better career progression and higher salaries (36). Extending OSH training to law enforcement agencies and the broader Malaysian population can enhance OSH from consciousness to full compliance.

Both groups identified challenges in training, reporting structures, organisational mindsets, public perception issues, social media management approval and the need for resilient leadership in addressing these challenges (37).

### OSH Enforcement and Organisational Reporting Structure

Group 2 expressed concerns about private security divisions enforcing OSH laws without government oversight, relying on self-regulation and company-specific rules to issue penalties by SHO, which collaborates with security officers (33). They also highlighted inconsistencies in OSH enforcement, such as assigning FSP despite Malaysian legislation not allowing it (2). They suggested AI-powered minor summons systems for OSH compliance, while Group 1 highlighted the positive impact of the automated enforcement system (38, 39).

### Industry and SME Compliance with the SSHE Model

The SSHE model was preferred by KIs due to its effectiveness in enhancing compliance, improving company revenues and enhancing reputations. Department of Occupational Safety and Health has mentored 1,175 manufacturing enterprises, but reaching all SMEs remains a challenge (40).

The SSHE model aims to improve worker and SME compliance, but effective awareness campaigns are needed to manage perceptions, prevent resistance and address misinformation (41, 42). The SSHE model’s success relies on legislative support, organisational commitment and worker participation (18, 33) supported by theoretical frameworks including ORCT (18, 43, 44), leadership (45), compliance (46) and enforcement (47).

### Limitations and Recommendations for Future Research

Studies on integrated support enforcement in Malaysia and elsewhere are scarce. In the UK, CSOs have similar low-level responsibilities, limited security authority, limited authority to issue fines, stop vehicles and investigate unethical behaviour (47). CSOs are responsible for enforcing security-related offences, not OSH.

As the study’s focus was on enforcement management, future research should use mixed methods to explore fine system enforcement, OSHA 1994 self-regulation, summons conduct factors and larger demographics (33, 47).

## Conclusion

The findings of this research, obtained through FGDs, revealed a consensus on the general objective of this study to establish the SSHE model by assessing the acceptance of the concept, challenges and perceptions of the new integrated roles of SSHEs. Most KIs from both groups agreed that Malaysia should adopt the SSHE model. The second objective was to enable legislation, organisations and workers to evaluate the adequacy of training modules for AP, RELA and security guards, and to benchmark current OSH coordinator modules to develop new modules (2, 11). According to all KIs, training is essential for law enforcement personnel to acquire and apply OSH knowledge in the workplace. There is a need for basic OSH training to be included in basic SP training, which could then be expanded to include other law enforcement agencies and all Malaysians. The SP training module was not included in any OSH curriculum. National standard training is needed (31).

As most KIs highlighted, this research is beneficial because it improves understanding of the impact of OSH on health and safety, raises awareness of diseases and hazardous practices, encourages readiness to change, and maximises organisational resources (18, 28, 48). While doing so, it controls costs to avoid burdening organisations. It also highlights the necessity of more robust enforcement measures and strong leadership to deter companies from endangering the health and safety of workers through poor OSH management systems (14, 45). It also emphasises the importance of skill levels for job promotion. The public and private sectors can impose minor fixed penalties using OSHA 1994 self-regulation with management approval. The third objective is to develop a new framework for this integrated model (Figure 2). If implemented, the study’s findings will contribute to the goals of SDG 8, and OSHMP30 will contribute to upholding the law by strengthening enforcement at the working level with minor fixed penalties.

## Figures and Tables

**Figure 1:**
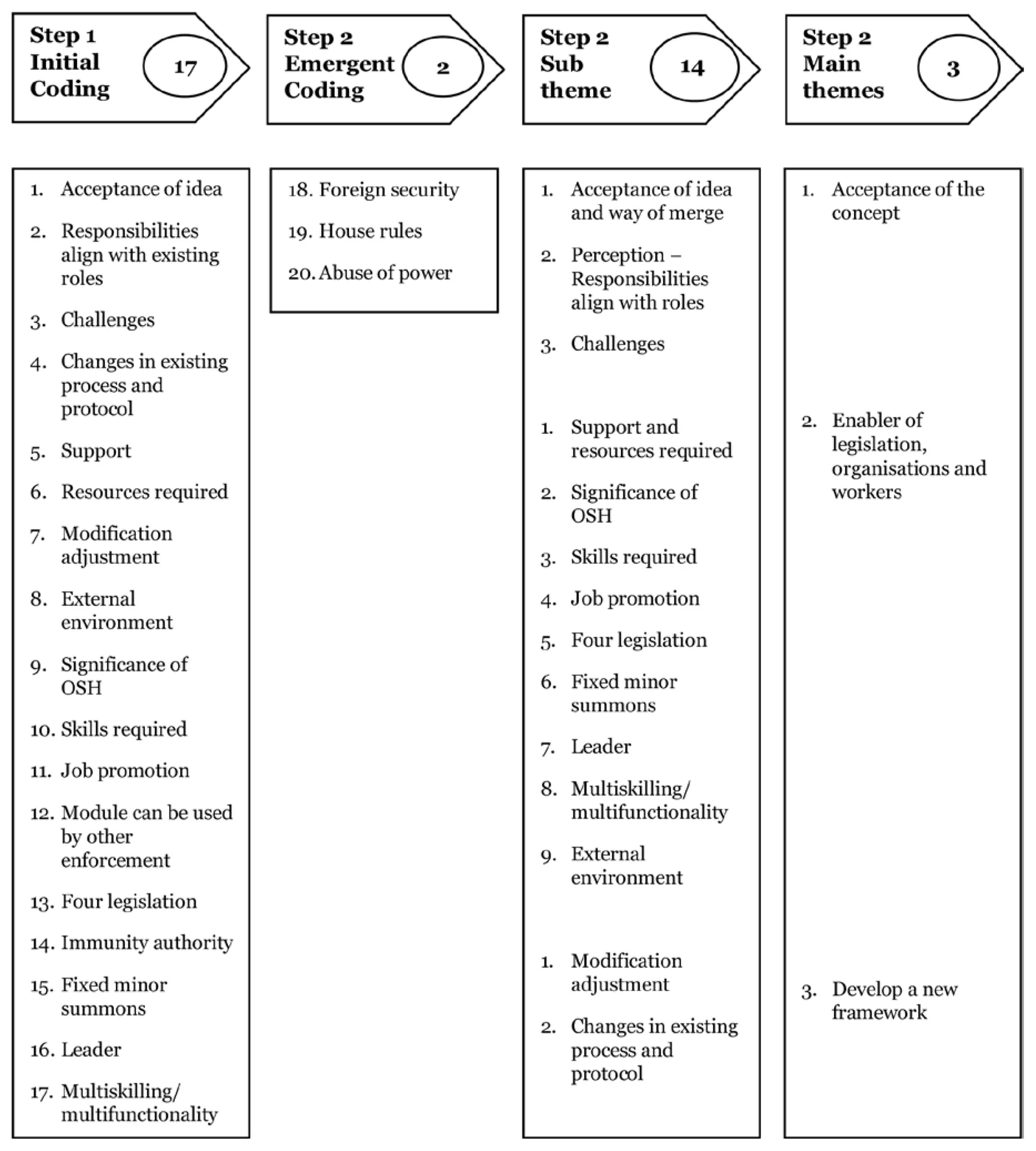
Framework analysis performed to identify themes.

**Figure 2:**
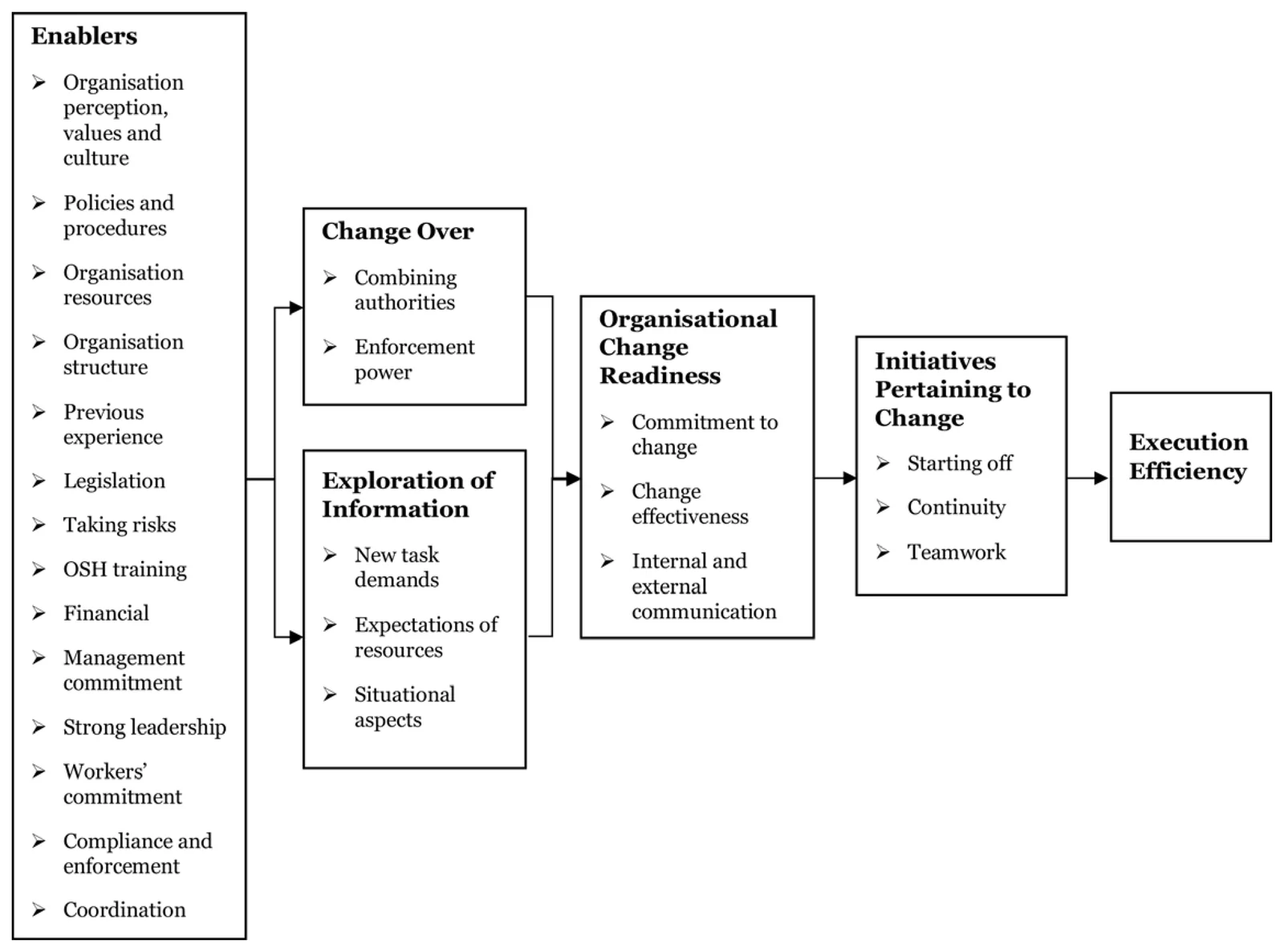
Enabler framework for SSHE roles developed using grounded theory analysis, FGD and ORCT.

**Table 1:** Sociodemographic information of focus group attendees (*N* = 11).

Variables	Outcome	
	Participated *n* (%)	Mean
**Both groups**		

Attendees	11 (100)	
FGD 1	5 (45)	
FGD 2	6 (55)	

Age		47
FGD 1		46.4
FGD 2		57.0

Experience		23.7
FGD 1		21.8
FGD 2		25.5

**Group 1**		

Age		46.4

Occupation		
Government officer	3 (60)	
Deputy CEO	1 (20)	
Director	1 (20)	

Work experience (years)		21.8
1 to 10	0 (0)	
11 to 20	3 (60)	
21 to 30	0 (0)	
31 to 40	2 (40)	
41 to 50	0 (0)	

Gender		
Male	3 (60)	
Female	2 (40)	

Race		
Malay	5 (100)	
Chinese	0 (0)	

**Group 2**		

Age		57.0

Occupation		
General manager	1 (16.7)	
Senior consultant	1 (16.7)	
NGO officer	4 (66.6)	

Work experience (years)		21.8
1 to 10	0 (0)	
11 to 20	3 (50)	
21 to 30	0 (0)	
31 to 40	2 (33.3)	
41 to 50	1 (16.7)	

Gender		
Male	6 (100)	
Female	0 (0)	

Race		
Malay	5 (83.3)	
Chinese	1 (16.7)	

FGD = focus group discussion; CEO = chief executive officer; NGO = non-governmental organisation
